# Bioelectronics for *In Situ* Monitoring of Tumor Microenvironment Markers

**DOI:** 10.7150/ntno.126464

**Published:** 2026-02-11

**Authors:** Kuldeep Mahato, Girijesh Kumar Patel

**Affiliations:** 1 Department of Biotechnology, Motilal Nehru National Institute of Technology Allahabad, Prayagraj, Uttar Pradesh, 211004, India.; 2 Cancer and Stem Cell Laboratory, Department of Biotechnology, Motilal Nehru National Institute of Technology Allahabad, Prayagraj, Uttar Pradesh, 211004, India.

**Keywords:** tumor microenvironment, bioelectronic sensors, *in situ* monitoring, electrochemical biosensing, molecular imaging, nano-contrast agents

## Abstract

The tumor microenvironment (TME) is a dynamic and heterogeneous niche that critically shapes cancer progression, immune evasion, and therapeutic resistance. Characterized by gradients in oxygen tension, pH, and metabolic activity, the TME offers a rich yet underexploited source of real-time biomarkers related to cancer. While conventional imaging techniques often lack the temporal resolution and molecular specificity to capture these rapid physiological changes, emerging miniaturized bioelectronics and multiplexed systems carry promise for *in situ* monitoring of such TME markers with high sensitivity and spatial precision. This article explores such recent advances in this direction, including bioelectronic sensor design, flexible electrochemical devices, organic transistors, and nanostructured interfaces tailored for TME characterization. Further, a discussion of the convergence of bioelectronics with nano-contrast-based molecular imaging is presented, with prospects for developing closed-loop therapeutic systems for cancer. These technologies offer a transformative platform for precision oncology, enabling dynamic, continuous, and localized assessment of tumor biology across preclinical and clinical settings.

## Introduction

Cancer is increasingly understood not as a disease of autonomous malignant cells alone, but as a systemic, multifactorial disorder shaped by complex interactions between tumor cells and their surrounding microenvironment 1-3. It is recognized as a highly dynamic and interactive ecosystem composed not only of malignant cells, but also of cancer-associated fibroblasts, infiltrating immune cell populations (including T cells, macrophages, dendritic cells, and natural killer cells), endothelial and stromal components, and diverse extracellular matrix (ECM) structures 4. Together, these constituents form a dynamic, adaptive niche that plays a decisive role in all stages of tumor development, from early neoplastic transformation to metastasis, immune evasion, and therapeutic resistance. The recognition that the TME serves not merely as a passive spectator but as an active participant in tumor biology has catalyzed a paradigm shift in how cancer is studied and treated 5. In particular, recent research has underscored the spatial and temporal heterogeneity of the TME, wherein gradients of oxygen, pH, reactive oxygen species (ROS), and metabolic by-products coexist with immunological and mechanical signals. These gradients are not static; rather, they evolve in response to cellular proliferation, therapy, immune surveillance, and angiogenesis 6. This continuous flux profoundly influences how tumors grow, interact with the host immune system, and respond, or fail to respond to treatment. Among the most salient features of the TME is hypoxia, a condition of reduced oxygen availability that arises due to abnormal and inefficient tumor vasculature 7. Hypoxic zones within tumors foster genomic instability, drive angiogenic signaling, alter cellular metabolism, and promote a more aggressive, therapy-resistant phenotype. Concurrently, the metabolic rewiring of cancer cells, exemplified by aerobic glycolysis and lactate accumulation, leads to extracellular acidosis, creating pH gradients that further sculpt the TME. These physicochemical stressors are compounded by elevated levels of ROS, which can act as signaling molecules at moderate concentrations but become cytotoxic and mutagenic at high levels 8. In parallel, immune cell infiltration into the TME is often co-opted by the tumor, leading to immune suppression through the secretion of cytokines, the expression of immune checkpoint molecules, and the recruitment of regulatory T cells and myeloid-derived suppressor cells 9. Together, these elements define a biochemical and cellular landscape that is both hostile to therapeutic efficacy and rich in diagnostic information.

Despite the clinical relevance of these TME features, capturing their dynamic behavior *in vivo* remains a long-standing problem. Conventional imaging modalities such as magnetic resonance imaging (MRI), positron emission tomography, computed tomography (CT), and even advanced optical techniques offer powerful anatomical and functional readouts, but they are limited in spatial resolution, molecular specificity, or temporal fidelity 10. These limitations are especially pronounced when seeking to monitor fluctuations in biochemical markers, such as pH or oxygen tension, on the timescales relevant to drug action or immune engagement 11,12. Furthermore, many imaging techniques rely on exogenous contrast agents administered systemically, which may not reflect localized or transient changes in the TME 13. Hence, there exists a critical need for next-generation technologies capable of real-time, *in situ* monitoring of TME markers with high sensitivity, specificity, and spatiotemporal resolution. Bioelectronic sensors have emerged as promising tools to fill this gap 14,15. These devices integrate electronic transducers, such as field-effect transistors, electrochemical electrodes, or organic semiconductors, with biological or chemical recognition elements, enabling the direct conversion of biochemical or physiological signals into quantifiable electronic readouts 16. Advances in materials science, micro- and nanofabrication, and wireless communication technologies have propelled recent progress in this field. As a result, bioelectronic sensors are now being developed in soft, stretchable, minimally invasive, and, in some cases, fully implantable or wearable formats 17,18. These innovations have enabled researchers to deploy sensors within or adjacent to tumors, allowing them to monitor local concentrations of oxygen, pH, ROS, metabolites such as glucose or lactate, and even immune-related biomarkers 15. Notably, bioelectronic systems can achieve continuous or high-frequency sampling, making them well-suited for longitudinal studies and therapeutic monitoring. In preclinical models, sensors have been integrated with tumor spheroids, 3D organoids, and orthotopic tumor implants to provide functional readouts that correlate with disease progression and treatment response. In some cases, these platforms have been extended to include closed-loop systems in which sensor feedback informs drug delivery or photodynamic therapy, thus transforming passive monitoring devices into active participants in the therapeutic process 19. The incorporation of nanomaterials has further enhanced the capabilities of bioelectronic devices 20-23. Nanostructured electrodes made up of carbon nanotubes, graphene, or conductive polymers offer high surface area, excellent charge-transfer properties, and the ability to immobilize functional biomolecules with high stability, contributing to improved sensitivity and selectivity, even in complex biological fluids such as interstitial tumor fluids or blood. Moreover, these nanoelectrodes enable multi-analyte detection, allowing a single device to simultaneously track multiple TME parameters and thus provide a more holistic view of tumor physiology 24.

In addition, a particularly exciting frontier lies in integrating bioelectronics with nano-contrast-based molecular imaging. Contrast agents, such as pH-sensitive nanoparticles, oxygen-sensitive fluorophores, or ROS-responsive probes, are coupled with bioelectronic devices, creating multimodal platforms that combine spatial imaging with real-time sensing 25. These hybrid systems promise to overcome the limitations of standalone imaging or sensor modalities, enabling the capture of the spatiotemporal biochemical state of tumors and their proximity using a miniaturized bioelectronic device. Such platforms could provide unprecedented insights into tumor heterogeneity, therapy-induced remodeling, and emergent resistance pathways.

In this article, we explore the rapidly evolving field of bioelectronics as applied to *in situ* monitoring of TME markers. We begin by surveying the key molecular and physiological signatures of the TME, highlighting their relevance to cancer biology and therapy. We then examine recent advances in bioelectronic device architectures, focusing on implantable, wearable, and organoid-integrated systems for monitoring oxygen tension, pH, redox states, and metabolic flux. Finally, we consider the convergence of bioelectronics with nano-contrast-enhanced imaging techniques, and the emerging opportunities and how this synergy presents molecular diagnostics, precision oncology, and real-time therapy guidance. Together, these developments point towards a new era in cancer research, one in which the TME can be monitored not just periodically, but continuously, accurately, and *in vivo* settings. In the following section, various such biomarkers for TME have been discussed.

## Key Markers in the TME and Sensing Requirements

**Figure 1:**
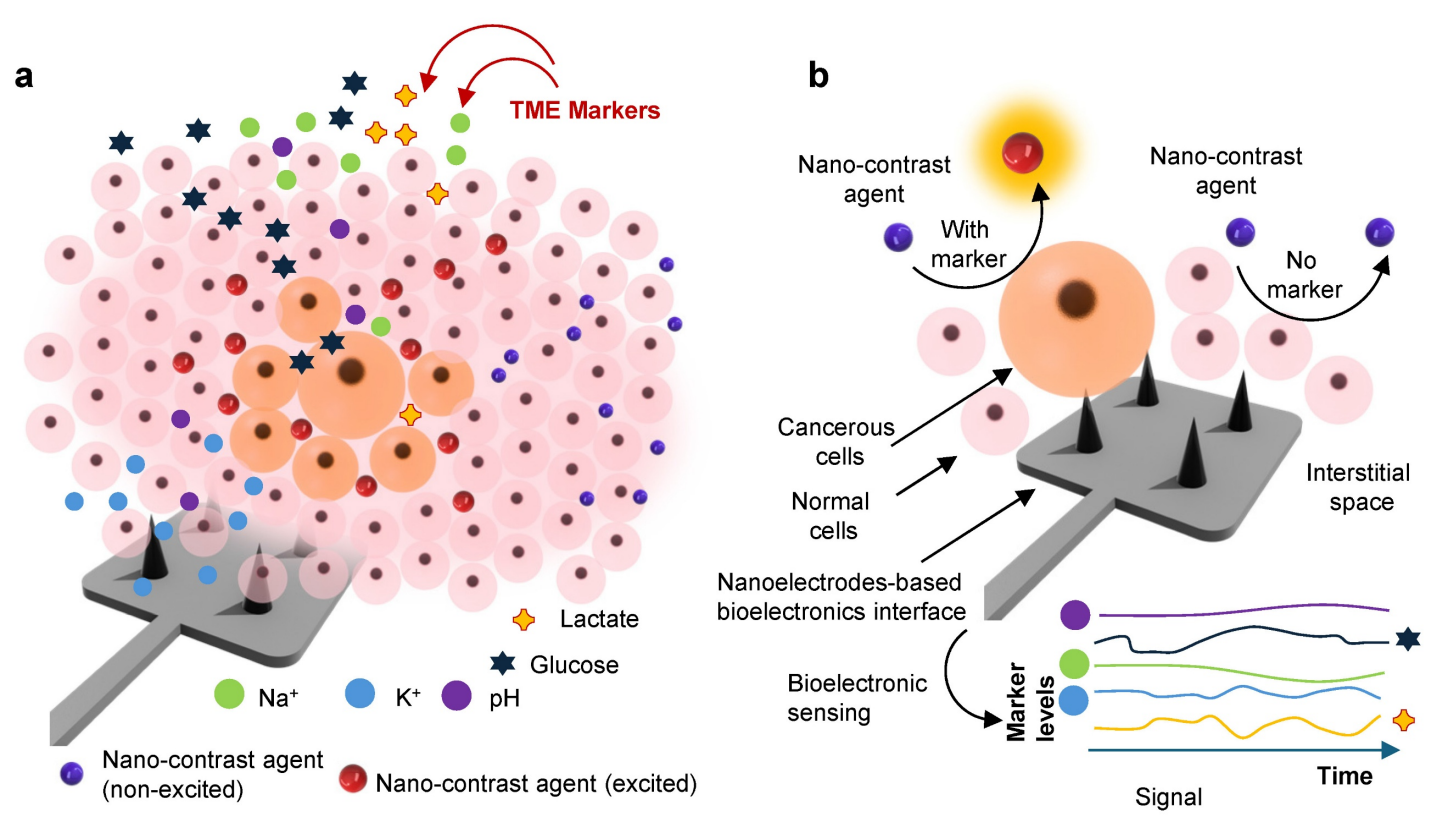
Mapping of TME using bioelectronic sensors and nano-contrast-agent based imaging: a. A schematic illustration represents the heterogeneous TME landscape, highlighting spatial gradients of biochemical markers, including metabolites (glucose, lactate), pH, ion concentrations (K^+^ and Na^+^), and other species, *viz.* peroxide, ROS, etc. b. A conceptual depiction of the orthogonal sensing paradigm where bioelectronic sensors capture real-time electrochemical signatures of TME marker dynamics, while nano-contrast-agent-enhanced imaging modalities visualize structural and molecular changes in depth. Together, these orthogonal sensing enable multimodal spatial-temporal mapping, improving tumor stratification, treatment planning, and longitudinal response monitoring.

The TME presents a complex biochemical and biophysical landscape, shaped by dynamic interactions among malignant cells, stromal components, and host immune elements. These interactions generate a set of hallmarks, hypoxia, acidosis, oxidative stress, metabolic reprogramming, immune modulation, and mechanical stress, that not only sustain tumor growth but also serve as functional biomarkers for disease stratification, therapy selection, and real-time response monitoring 7. Figure 1a illustrates the TME landscape releasing various markers upon receiving cellular and subcellular stress. Detecting these signatures *in situ* and continuously, however, demands sensor platforms capable of interfacing intimately with the tissue microenvironment. As bioelectronics mature to meet this challenge, it is imperative to understand both the biological underpinnings of TME markers and the sensing requirements that determine their effective *in vivo* capture 26.

### Oxygen Tension and Hypoxia

One of the most extensively studied parameters in TME is oxygen tension. Hypoxia arises as a consequence of chaotic and insufficient vascularization, leading to regions within tumors where oxygen partial pressures (pO₂) fall well below physiological requirements 27. These hypoxic niches are non-homogeneous and exist as fluctuating gradients influenced by vascular density, perfusion pressure, and metabolic consumption. Hypoxia stabilizes hypoxia-inducible factors, which in turn drive transcriptional programs associated with angiogenesis, immune suppression, metabolic adaptation, and therapy resistance 28. Measuring oxygen tension in real-time within the TME is therefore crucial not only for understanding tumor aggressiveness but also for predicting response to therapies such as radiotherapy, where efficacy is largely dependent on oxygen 29. Electrochemical oxygen sensors, particularly Clark-type electrodes and solid-state potentiometric devices, offer direct quantification of pO₂ with sub-second temporal resolution 30,31. However, effective deployment *in vivo* requires miniaturization, minimal oxygen consumption by the sensor itself, and calibration against reference standards. Furthermore, as oxygen gradients vary over micrometer-length scales, spatially resolved sensor arrays or sensor-integrated mapping techniques are often necessary to generate interpretable profiles of tissue oxygenation.

### pH and Acidosis

In addition to hypoxia, extracellular acidosis is a hallmark of TME. Cancer cells preferentially utilize glycolysis even under normoxic conditions due to consistent energy demand, a phenomenon known as the Warburg effect, resulting in the accumulation of lactic acid and protons in the extracellular space 32. The resulting acidic milieu, with pH often dropping below 6.5, promotes ECM degradation, modulates immune cell behavior, and impairs drug efficacy by altering uptake and solubility. Accurate sensing of extracellular pH poses several challenges. pH is a logarithmic scale, meaning that small changes in proton concentration reflect exponential shifts in acidity. Additionally, pH can be locally buffered by proteins, bicarbonate, and matrix interactions, requiring sensors to respond selectively to free proton concentration while resisting drift from nonspecific interactions. Ion-sensitive field-effect transistors, potentiometric microelectrodes, and redox-based pH indicators have demonstrated promise in this space. Integration with organic materials, such as pH-responsive conducting polymers, has enabled enhanced flexibility and tunability. For *in vivo* applications, the sensor must preserve responsiveness while allowing rapid ionic exchange, and calibration protocols must compensate for temperature, ionic strength, and tissue heterogeneity 14.

### Reactive Oxygen Species (ROS) and Redox Balance

The ROS, including hydrogen peroxide, superoxide anions, and hydroxyl radicals, play dual roles in cancer progression. At controlled levels, ROS act as secondary messengers modulating proliferation, angiogenesis, and immune signaling. At higher concentrations, they induce oxidative damage and apoptosis 33. Elevated ROS levels within the TME are often linked to mitochondrial dysfunction, inflammatory cell activity, and radiation-induced oxidative stress 34. Capturing redox fluctuations *in vivo* is technically demanding due to the short half-lives and diffusivity of many ROS species. Traditional approaches rely on fluorescent dyes or chemiluminescent probes that undergo oxidative cleavage or structural rearrangement. However, these techniques are not easily adapted for real-time or longitudinal measurements 35. Bioelectronic strategies, especially those employing redox-active surfaces or enzyme-functionalized electrodes, offer a promising alternative. For example, sensors functionalized with horseradish peroxidase or manganese-based catalysts can transduce ROS-mediated redox events into electrical signals 36. However, specificity remains a concern, as ROS often coexist with other reactive nitrogen species and metabolites. Furthermore, the sensors must avoid self-degradation or signal interference during extended deployment, requiring robust surface passivation and reference-channel calibration.

### Metabolites and Energy Status

The metabolic signature of the TME is another key axis for sensor development. Tumor cells exhibit enhanced uptake of glucose, increased secretion of lactate, and elevated turnover of ATP and NADH, all of which create measurable gradients in metabolite concentrations. These gradients are intimately tied to hypoxia and pH, but also provide distinct insights into the energetic and biosynthetic status of cancer 37. Electrochemical biosensors targeting glucose and lactate have achieved significant clinical translation in other domains, such as diabetes monitoring and sports physiology. These platforms typically use immobilized enzymes (glucose oxidase or lactate oxidase) to generate redox-active species proportional to substrate concentration 38. When reconfigured for implantation in the tumor and surrounding tissues, these sensors must overcome challenges of fouling, motion artefacts, and matrix interactions. The development of non-enzymatic metabolite sensors, using metal-organic frameworks, nanowires, or catalytic surfaces, offers an exciting direction for achieving enzyme-independent sensing with higher operational stability.

### Immune Signaling Molecules and Cytokines

While chemical gradients such as oxygen and pH are relatively well characterized, the molecular signals that govern immune dynamics in the TME are only beginning to be integrated into real-time sensing platforms. Cytokines such as interleukin-6, tumor necrosis factor-alpha (TGF-α), and transforming growth factor-beta (TGF-β), along with immune checkpoint ligands like PD-L1, orchestrate the immune tone of the tumor 39. These biomarkers are often present at picomolar concentrations and fluctuate rapidly in response to immune infiltration or therapy. Sensing such low-abundance proteins requires platforms with high sensitivity and selectivity. Field-effect biosensors functionalized with aptamers, antibodies, or molecularly imprinted polymers have shown potential for label-free, multiplexed detection 40. However, their integration into implantable or wearable formats remains nascent. Key barriers include nonspecific binding in protein-rich environments, instability of biorecognition elements, and difficulty in achieving regeneration or recalibration of the sensing interface 41.

### Mechanical and Ionic Microenvironment as TME markers

Beyond chemical cues, the TME is defined by abnormal mechanical properties and ionic fluxes. Increased matrix stiffness altered tissue viscoelasticity and elevated interstitial fluid pressure influence cell motility, phenotype, and response to treatment 42. Additionally, ion channel activity, particularly involving calcium, potassium, and chloride, modulates cancer cell proliferation and immune cell recruitment. Recent advances in soft electronics have enabled the design of strain-sensitive and pressure-responsive sensors that can interface with Tumors and report mechanical properties *in vivo* 43. Simultaneously, ion-sensitive bioelectronic devices capable of tracking fluctuations in calcium or potassium levels are being developed, particularly in the context of neuro-oncology and tumor-associated seizure disorders 44,45.

While these markers have commonly been targeted towards monitoring TME using various biomolecular techniques, the adoption of bioelectronics would leverage efficient and precise quantification of these markers *in situ* settings. The following section discusses such advances in the development of bioelectronics for *in situ* monitoring of TME markers.

### Recent Advances in *In Situ* Bioelectronic Monitoring of TME Markers

In recent years, bioelectronic sensors have moved from proof-of-principle demonstrations in simple *in vitro* settings toward more realistic, *in vivo*, and even large-animal models 46. These advances have been enabled by the monitoring systems (Figure 1b) and their integration with devices 47,48. This section discusses various state-of-the-art techniques of TME marker monitoring and their general trends employing bioelectronics.

One of the most significant milestones is the development of miniature implantable electrochemical oxygen sensors for monitoring tumor hypoxia. In a study, Marland et al. implanted a microfabricated oxygen sensor into naturally occurring lung tumors in sheep. The device was fabricated on a silicon substrate with an integrated reference electrode and electrolyte membrane. It exhibited a linear response to oxygen concentration, was robust to sterilization and irradiation, and maintained function after CT-guided implantation in the tumor tissue. Importantly, it could detect acute changes in oxygenation in response to physiological perturbation. While bioelectronics is sensitive, there have been some limitations regarding the issue of susceptibility to biofouling and drift over time 29. Another recent advance is the design of wireless, ultrasonic-powered implants for tissue oxygen monitoring. In other words, a tiny implant (<5 mm) that combines a micro-LED, oxygen-sensitive film, and optical detector, paired with a piezoelectric component to transmit data via ultrasound. In sheep, this implant processed oxygen signals in muscle tissue, encoding them via backscatter ultrasound and enabling external readout. Although not yet deployed in tumor tissue, the platform could, in principle, be used for tumor hypoxia monitoring, especially for deep-lying lesions where optical access is limited. Crucially, the device demonstrated ten-day stability in human serum and showed that miniaturization and wireless operation are possible without severely compromising the sensitivity 49. Meanwhile, in the domain of pH and metabolite sensing, flexible, wearable, and implantable biosensors have made strides. Nguyen et al. have developed a subcutaneous sensor capable of continuously monitoring both pH and lactate levels *in vivo*. This photonic sensor (PALS) uses optical detection (luminescence or fluorescence) modulated by biochemical probes sensitive to lactate and proton concentrations, enabling dynamic tracking of these markers in living tissue. Such dual parameter sensing is valuable because lactate accumulation and acidosis often proceed together under hypoxia, but can also diverge depending on perfusion, metabolic adaptation, or therapy 50. In parallel, rapid progress has been made in the development of engineered organic electrochemical transistors (OECTs), an emerging class of mixed ionic-electronic conducting devices derived from organic field-effect transistor architectures. OECTs exploit volumetric doping and de-doping mechanisms within conductive polymers, most notably PEDOT: PSS, to transduce biochemical events into amplified electrical signals. Recent advances have enabled the design of OECT platforms capable of detecting a broad spectrum of analytes, ranging from small metabolites (e.g., glucose, lactate) and key neurotransmitters (e.g., dopamine and serotonin) to higher-order macromolecular biomarkers, including proteins, hormones, and nucleic acids. Their inherent mechanical softness, excellent biocompatibility, high transconductance, and ability to operate at ultralow voltages collectively position OECTs as promising candidates for long-term *in vivo* and chronic implantation applications. These features are particularly advantageous for interfacing with soft biological tissues such as neural and cardiac systems, where conventional rigid electronics often fail due to inflammation, signal drift, or mechanical mismatch. For example, some OECT devices employ enzyme functionalization (e.g., lactate oxidase) or redox mediators, coupled with nanostructured electrodes (graphene, metal nanowires) or conductive polymers (e.g., PEDOT: PSS) to improve sensitivity 51. Another area of progress is electrochemical detection of ROS, particularly hydrogen peroxide (H₂O₂) 45. These sensors use immobilized peroxidase enzymes, catalytic nanomaterials (e.g., metal oxides, nanozymes), or redox-active electrodes to transduce ROS into electrical signals. While many are tested in buffer or cell culture, a subset is being pushed toward *in situ* or *in vivo* applicability. Table 1 summarizes various feasible strategies of bioelectronic sensors for cancer environment detection, which can be miniaturized for developing the TME monitoring. Although these bioelectronic sensors offer better analytical performances, the challenges include interference from other oxidants, drift, and ensuring fast response/clearance 52.

A more integrative case is the embedding of sensor particles into 3D tumor models or tumor-stromal co-cultures, where gradients of oxygen, pH, and metabolites develop spontaneously. For instance, fluorescent or phosphorescent silica microparticles, doped with ratiometric dyes, have been used to map oxygen and pH gradients in 3D scaffolds or tumor spheroids 53. These provide spatial resolution (via imaging) and allow correlation of sensor outputs with cell viability, gene expression, or therapy response. Though not strictly bioelectronic, such systems inform the design of bioelectronic devices by revealing the scale and heterogeneity of gradients that devices must resolve. Taken together, these examples illustrate key enabling features of recent advances. First, anatomical guidance and imaging modalities are increasingly used to precisely position sensors, ensuring readings are meaningful in tumor tissue rather than peritumoral or normal tissue. Second, wireless or minimally invasive implants reduce the burden of tethering and improve the feasibility of longitudinal studies. Third, dual or multiplexed sensing (oxygen + pH, lactate + pH, etc.) is beginning to emerge as a crucial system for interpreting TME dynamics, since single markers often do not paint the full picture (e.g., low oxygen but good perfusion might mitigate some effects of hypoxia). Fourth, the use of biocompatible materials and soft, flexible device architectures helps to reduce tissue damage, immune response, and mechanical mismatch, improving signal stability over time. Nevertheless, biofouling, the adhesion of proteins, cells, or fibrous tissue to sensor surfaces, continues to degrade their performance, especially in long-term implantation.^54^, ^55^ Drift due to material aging, changes in electrode surface chemistry after sterilization, or encapsulation issues undermines quantitative reliability 56. Integration into deeper tissues (e.g., pancreatic, brain, liver) remains difficult due to issues of power delivery, communication, and biocompatibility. Further, for many electrochemical or optical probes, selectivity in the presence of co-existing redox species, variable ionic strength, and temperature fluctuations remains a challenge, and these can all introduce noise or bias 57.

As the field advances, combining nano contrast agents with bioelectronic devices appears promising. For example, optical or fluorescent contrast agents whose emission is modulated by local oxygen or pH can be integrated with electrochemical transducers for orthogonal read-out 58. These orthogonal sensors will not only enable accurate real-time profiling of the TME environment but also offer spatial resolution. In the following section, we have discussed various such strategies exploiting their synergy.

## Engineering Convergence: Tumor Progression, Bioelectronic Innovations, and Synergy with Molecular Imaging

Tumor progression represents a dynamic and multifactorial biological process involving genetic mutations, epigenetic drift, metabolic rewiring, immune evasion, and crosstalk with the surrounding TME 2. As cancers evolve from localized, indolent lesions into invasive, angiogenic, and metastatic phenotypes, the underlying biochemical and biophysical landscapes undergo dramatic changes 59.

Traditional histopathology and biochemical assays, though indispensable, offer static snapshots that cannot capture the real-time transitions in extracellular matrix composition, hypoxia gradients, metabolic flux, immune infiltration, or vasculature function 60. To interrogate this evolving complexity, modern oncology increasingly demands convergent engineering: an integrated framework where bioelectronic sensors, multimodal imaging platforms, and molecular diagnostics operate synergistically to provide continuous, high-resolution information on tumor behavior 61. The intersection of bioelectronics, molecular imaging, and cancer biology is ushering in a new era of precision oncology in which tumors are no longer viewed as opaque entities but as dynamic, measurable systems with quantifiable electrical, biochemical, and metabolic signatures 62. This engineering convergence transcends conventional siloed approaches by integrating real-time biophysical sensing with spatially resolved imaging modalities, bridging millimeter-scale anatomical visualization with microscale and nanoscale molecular sensing.

Tumor progression as a multiscale bioelectronic phenomenon: Tumor progression is increasingly recognized as a phenomenon that unfolds across multiple biological, chemical, and electrical scales 63. While conventional oncology focuses on genetic mutations, aberrant signaling cascades, and dysregulated metabolism, an emerging perspective highlights that cancer fundamentally rewires the physicochemical and bioelectronic landscape of tissues. These electrical and ionic perturbations are not passive reflections of malignancy; rather, they actively contribute to tumor initiation, growth, invasion, and therapeutic resistance 64. A defining feature of cancer cells is the persistent depolarization of their plasma membranes. Normal differentiated cells maintain a highly negative resting membrane potential, whereas cancer cells exhibit a more depolarized state driven by altered expression of voltage-gated sodium, calcium, and potassium channels 65. This depolarized profile correlates strongly with proliferative drive, stem-like phenotypes, and epithelial-mesenchymal transition 66. Ionic channels, once viewed as simple conductance regulators, now emerge as oncogenic hubs, where elevated K⁺ efflux facilitates cytoskeletal remodeling and invasive migration, while dysregulation of chloride channels shapes cell volume changes essential for metastasis 67. At the tissue level, tumor expansion disrupts ion homeostasis, generating abnormal extracellular K+ accumulation and spatial electrical gradients that further modulate immune cell infiltration and stromal activation 68.

Simultaneously, the evolving TME becomes a unique electrochemical niche. Hypoxia, driven by chaotic angiogenesis, shifts tumor metabolism toward aerobic glycolysis, resulting in excessive lactate production and acidification 69. Redox-active species such as NADH/NAD⁺, glutathione, and ROS accumulate, producing distinct electrochemical signatures that reflect metabolic rewiring and oxidative stress. As interstitial fluid pressure increases and ECM components become cross-linked or charged, the conductivity and dielectric properties of the tumor tissue shift markedly, alterations that can now be sensitively probed using emerging bioelectronic tools 70. These physicochemical transformations provide fertile ground for next-generation biosensing. Implantable microelectrodes and flexible organic electrochemical transistors can detect subtle shifts in redox metabolites, pH, and ionic flux in real-time 71. Minimally invasive microneedle platforms integrated with multiplexed electrochemical sensors capture oxygen gradients, cytokine surges, and metabolic precursors directly from interstitial fluid. By translating these biochemical and electrical cues into quantifiable signals, bioelectronic devices offer continuous monitoring of tumor evolution at unprecedented resolution. Thus, tumor progression should be viewed through a dual lens: biological and electrochemical. Integrating bioelectronic sensing with imaging, liquid biopsy, and omics-driven diagnostics creates a powerful complementary axis capable of revealing early malignant transformation, forecasting therapeutic response, and enabling precision oncology interventions 72.

Bioelectronic innovations for *in vivo* tumor sensing: The landscape of tumor diagnostics has undergone a profound transformation with the rise of bioelectronic technologies capable of continuously interrogating the TME. Unlike conventional imaging or blood-based assays that provide intermittent information, these soft and miniaturized electronic interfaces enable real-time, dynamic surveillance of tumor physiology 11. By integrating seamlessly with living tissue, they capture subtle biochemical, electrical, and metabolic fluctuations that define tumor evolution, thereby establishing a new paradigm for precision oncology 73.

Among the most impactful innovations are OECTs, which leverage the mixed electronic-ionic conductivity of materials such as PEDOT: PSS to interface intimately with soft biological tissues. Their volumetric mode of operation allows them to detect minute changes in ion transport, redox state, and electrophysiological activity induced by cancer progression 74. When implanted adjacent to or within tumors, OECTs can record pH fluctuations driven by glycolytic metabolism, monitor oxidative stress through ROS-responsive channel activity, and sense variations in ion gradients associated with cellular proliferation, angiogenesis, and extracellular matrix remodeling. Their mechanical flexibility ensures long-term operation without provoking significant inflammatory responses, allowing chronic monitoring that captures the dynamic nature of the TME 75. Another major advancement lies in microneedle-based systems for tumor biomarker sensing. These minimally invasive platforms, constructed from solid metals, hollow polymers, or hydrogel matrices, penetrate only the superficial layers of tissue, enabling painless and frequent access to interstitial fluid 76. Microneedles can detect tumor-associated analytes long before they appear in systemic circulation. So far, these microneedles have been used to quantify lactate, inflammatory cytokines such as IL-6 and IL-8, angiogenic factors like VEGF, matrix-degrading enzymes including MMP-2/9, and even tumor-derived exosomes and cell-free DNA 77. In parallel, the development of flexible and bioresorbable electronics has enabled temporary implantation of monitoring devices during postoperative recovery or treatment cycles 78. Constructed from ultrathin biodegradable silicon, magnesium conductors, silk fibroin, and transient polymers, these systems conform to irregular tumor geometries and naturally dissolve after their monitoring window 79. They can track local inflammation, thermal changes during ablation therapies, healing processes, or residual tumor activity, eliminating the need for surgical removal.

Molecular imaging using nano-contrast- the spatial dimension of cancer monitoring: Molecular imaging enriched with nano-contrast agents has fundamentally transformed how tumors are visualized, monitored, and therapeutically evaluated 80. While bioelectronic sensors provide continuous, high-temporal resolution insights into biochemical fluctuations, imaging modalities augmented with engineered nanoparticles supply the missing spatial and anatomical context, revealing where, how, and to what extent these biological changes unfold within the tumor microenvironment. Nano-contrast agents amplify signal sensitivity, extend imaging depth, and introduce molecular specificity, allowing cancer to be mapped as a dynamic, spatially heterogeneous system 81. Nanoparticle-enabled positron emission tomography (PET) and single-photon emission computed tomography elevate functional imaging beyond traditional tracers like fluorodeoxyglucose and Fluoromisonidazole 82. Radiolabeled liposomes, polymeric nanoparticles, and iron oxide constructs demonstrate prolonged circulation, enhanced tumor accumulation via the enhanced permeability and retention effect, and active targeting of receptors involved in angiogenesis, hypoxia, and immune evasion. These platforms provide high-precision visualization of metabolic flux and inflammatory niches, signatures often invisible to small-molecule tracers.

In addition, MRI also gains extraordinary power through nano-contrast engineering. Superparamagnetic iron oxide nanoparticles, gadolinium nanoclusters, and manganese-based nanostructures enhance relaxivity, enabling clear delineation of micro-metastases, tumor margins, and regions of altered vascular permeability 83. Responsive nanoparticle systems that change signal profiles with pH, redox state, or enzymatic activity allow MRI to capture biochemical gradients aligned with tumor aggressiveness 84. When integrated with MR spectroscopy, metabolite-responsive nano-contrast agents amplify the detection of choline, lactate, and glutamine, adding metabolic mapping to spatial imaging 84. Optical and photoacoustic imaging further exemplifies the versatility of nano-contrast. Quantum dots, gold nanorods, and semiconducting polymer nanoparticles provide bright, multiplexed optical signatures for tracking protease activity, tumor margin evolution, and stromal remodeling 85. In photoacoustic imaging, plasmonic nanoparticles efficiently convert light to acoustic waves, enabling deep tissue profiling of oxygen saturation, angiogenesis, and hemoglobin dynamics with high resolution. Collectively, nano-contrast-enhanced molecular imaging delivers a multidimensional spatial map of tumor biology, allowing continuous biosensor data, such as lactate, pH, or cytokine fluctuations, to be accurately localized within the evolving tumor architecture. This integration establishes a comprehensive framework for precision oncology, where dynamic biochemical signals and spatial imaging converge to illuminate the full complexity of cancer progression.

Synergy between bioelectronics and molecular imaging for TME monitoring: The deepest transformation in cancer monitoring emerges not from bioelectronics or molecular imaging individually, but from their deliberate integration. When continuous biochemical sensing is unified with spatially resolved imaging, tumors can be interrogated across temporal, anatomical, and molecular scales simultaneously. This engineering convergence produces a multidimensional understanding of tumor biology, capturing rapid metabolic oscillations, mapping microenvironmental gradients, and contextualizing these fluctuations within the evolving three-dimensional architecture of cancer. It is this synergy that unlocks comprehensive, precision-driven monitoring strategies impossible for either modality alone. A central aspect of this integration is the co-registration of electrochemical sensor outputs with imaging datasets. Signals derived from microneedle arrays or implanted organic electrochemical transistors can be superimposed onto MRI, PET, or photoacoustic images, allowing biochemical and electrical fluctuations to be traced to precise anatomical structures. For example, spikes in lactate can be detected by microneedle sensors and mapped onto metabolically active tumor rims, while ROS fluctuations recorded by an OECT implant can be spatially aligned with fluoromisonidazole-PET-defined hypoxic niches. This alignment transforms abstract electrical data into physiologically interpretable patterns, revealing how biochemical dynamics relate to invasive fronts, necrotic cores, angiogenic regions, or stromal-immune interactions. Imaging can further guide the precise placement of sensors to maximize data relevance. Intraoperative ultrasound can localize fluid-rich, metabolically active pockets optimal for microneedle insertion; contrast-enhanced MRI identifies perfusion-altered zones where redox sensing may be most informative; fluorescence or Raman imaging highlights tumor margins or protease-active regions suitable for placing temporary or bioresorbable implants. This imaging-informed deployment ensures that sensors probe biologically meaningful microenvironments rather than random or uninformed tissue regions.

The synergy also operates in the reverse direction: bioelectric biomarkers help validate and refine imaging biomarkers. Continuous hypoxia sensing can confirm the biological relevance of fluoromisonidazole-PET signals; lactate flux measurements can strengthen MR spectroscopy findings; ion-channel-based electrophysiological shifts can corroborate diffusion-weighted MRI markers of tissue density. This cross-validation enhances diagnostic confidence and reduces ambiguity in interpreting imaging artifacts. The most powerful impact of this integration is its potential to guide closed-loop therapy. By merging the temporal precision of biosensing with the spatial insight of imaging, clinicians can design adaptive therapy strategies. Real-time lactate or cytokine elevations can trigger localized drug release monitored under MRI guidance; ROS sensing can inform radiation dose adjustments to exploit radiosensitivity windows; inflammatory profiles can dynamically schedule immunotherapy cycles. Over time, periodic imaging updates recalibrate the “map” while continuous sensing updates the “clock” enabling treatments that respond to tumor evolution as it unfolds. Together, bioelectronics and molecular imaging form a unified, synergistic framework that maximizes diagnostic depth, therapeutic precision, and biological insight, paving the way for truly adaptive, personalized oncology.

## Conclusions and outlook

As the biological understanding of the TME deepens, it becomes increasingly clear that its dynamic and heterogeneous landscape holds not only the key to cancer progression but also untapped potential for real-time diagnostics and precision-guided therapies. The development of bioelectronic systems tailored for *in situ* monitoring of TME markers represents a transformative step in this direction. Unlike conventional imaging modalities or *ex vivo* biomarker assays, bioelectronic platforms offer the capacity to sense molecular and physiological changes in real time, directly within the tumor niche 11,86. These devices enable high temporal resolution can be deployed chronically or during key therapeutic windows and when properly engineered allow for multiplexed readouts from a single miniaturized interface. Yet, the success of these platforms is not defined solely by technical prowess, but by their ability to align with the evolving demands of oncology. Cancer therapies are becoming increasingly personalized, with a focus on adaptive regimens that respond to biological feedback. The emergence of immunotherapies, metabolic inhibitors, and targeted agents has outpaced the development of tools to dynamically assess treatment response. In this context, bioelectronic sensors can bridge the gap, serving as molecular sentinels that report on treatment efficacy, tumor reprogramming, or emergent resistance in near real-time.

Despite significant progress, critical challenges remain to be addressed before these systems can be widely deployed in clinical oncology. Biocompatibility, stability, and regulatory approval are not trivial concerns. Implanted or tissue-integrated devices must operate in complex environments subject to immune surveillance, mechanical motion, and biofouling. Long-term stability of signal, reproducibility across patient populations, and standardization of calibration protocols will be essential for acceptance in translational and regulatory frameworks. Furthermore, data interpretation in the context of highly heterogeneous tumors will require not only robust electronics but also sophisticated algorithms capable of integrating multimodal signals into actionable insights. Ensuring long-term accuracy of bioelectronic sensors for TME marker monitoring remains a technical challenge due to signal drift, fouling, and fluctuating local conditions 87. Conventional calibration approaches, often performed *ex vivo* or during implantation, are insufficient for dynamic TME environments. To address this, several innovative strategies are emerging. Dual-channel reference designs now allow real-time differential measurements to internally correct for baseline drift or environmental interference. Self-calibrating biosensors, incorporating reversible redox probes or built-in standards, are being developed to autonomously recalibrate without external input 88. Furthermore, AI-assisted drift correction algorithms, trained on historical signal trends and physiological context, are beginning to demonstrate promise in compensating for baseline shifts that occur during chronic use 89. These smart calibration frameworks are particularly important for long-term systems operating without external recalibration access. As these technologies mature, they are expected to significantly improve the reliability, autonomy, and translational viability of *in situ* tumor biosensing platforms.

The next generation of bioelectronic platforms for *in situ* TME monitoring must move beyond static sensing toward integrated, intelligent systems capable of real-time adaptation and therapeutic feedback. While recent advances have demonstrated proof-of-concept devices for sensing oxygen, pH, ROS, and metabolic markers, significant challenges remain in achieving long-term biocompatibility, mitigating sensor drift, and ensuring calibration in heterogeneous tumor settings. Future efforts should prioritize closed-loop architectures that combine continuous biosensing with localized drug delivery or phototherapy, guided by AI-powered signal interpretation. Furthermore, hybrid platforms that merge bioelectronic readouts with nano-contrast-enhanced molecular imaging will enable a more comprehensive spatial and temporal mapping of TME dynamics. Finally, standardization in device fabrication, benchmarking protocols, and regulatory frameworks will be critical to ensure clinical translation. By addressing these needs, the field is poised to deliver bioelectronic tools that not only monitor but actively modulate tumor biology, ushering in a new era of precision oncology.

Looking forward, several areas hold promise for overcoming these challenges and expanding the scope of bioelectronic TME monitoring. First, innovations in self-healing and anti-fouling materials, including zwitterionic polymers and dynamic covalent hydrogels, may prolong device function and mitigate immune reactions. Second, the adoption of nanofabrication and 3D printing techniques offers pathways to create anatomically conformable sensors with high spatial resolution, capable of mapping local gradients across tumor borders or within invasive margins. These technologies will be instrumental in fabricating sensor arrays that match the architectural complexity of tumor tissues. Another exciting avenue is the integration of artificial intelligence and machine learning into bioelectronic systems. As sensors generate continuous streams of high-dimensional data, tracking changes in oxygenation, pH, lactate, and cytokine levels over time, AI algorithms can detect subtle trends, predict treatment failure, or even propose therapeutic adjustments. Such intelligent sensing systems could function as digital biomarkers, aiding clinicians in real-time decision-making or triggering alerts when physiological thresholds are crossed. Furthermore, the synergy with nano-contrast-enhanced imaging will likely define the next generation of hybrid diagnostic tools for tumor progression and therapeutic efficacy monitoring. The co-localization of molecular contrast agents, such as oxygen- or pH-responsive nanoparticles, with bioelectronic transducers enables multimodal sensing platforms that couple real-time monitoring with spatial imaging. These could be applied intraoperatively for tumor margin assessment or during therapy cycles to track microenvironmental normalization or exacerbation. Such dual-mode systems, if properly miniaturized and integrated with wireless communication modules, could offer a level of surveillance previously confined to experimental imaging suits. Equally important is the expansion of disease models and applications beyond oncology. While this perspective article focused on tumors, the foundational principles of bioelectronic monitoring, continuous, real-time sensing of dynamic biochemical environments, are directly applicable to inflammation, fibrosis, infection, and metabolic diseases. For example, tracking immune cell activation, tissue oxygenation, or metabolic shifts in autoimmune disorders or transplant rejection could leverage similar sensor platforms, expanding both clinical impact and commercial viability. Ultimately, the path forward will depend on collaborative ecosystems that bring together engineers, biologists, clinicians, and regulatory scientists. The translation of bioelectronic TME monitors from bench to bedside is not solely a technical journey but one that traverses patient safety, usability, economic feasibility, and ethical oversight. Cross-disciplinary consortia, shared device standards, and open-access datasets will accelerate progress toward scalable, interoperable, and clinically meaningful sensing platforms. In conclusion, bioelectronics for *in situ* monitoring of the TME is transitioning from a visionary concept to a practical, scalable frontier in precision oncology. By providing continuous, quantitative, and localized insights into the tumor niche, these devices have the potential to revolutionize how we diagnose, monitor, and treat cancer. As materials, electronics, and biological insights co-evolve, too will our ability to interface with disease, not merely to observe it, but to anticipate and actively shape its trajectory.

## Figures and Tables

**Table 1:** Bioelectronic sensing strategies those can be adopted for cancer environment sensing/monitoring.

Sensor Type	Target Marker(s)	Detection Mechanism	Key Advantages	Potential limitations
Electrochemical (Amperometric / Potentiometric)	Oxygen, pH, Lactate, Glucose	Direct redox reactions, potentiometric ion response	Simple fabrication; high temporal resolution; miniaturization	Susceptible to drift, biofouling, and calibration challenges
Organic Electrochemical Transistors (OECTs)	Lactate, Glucose, Cytokines	Conductivity modulation of organic semiconductors by ionic signals	High sensitivity; low operating voltage; flexible; multiplexing possible	Signal variability *in vivo*; biostability of organic materials
Redox / ROS Sensors	H₂O₂, Superoxide, Redox potential	Enzymatic or catalytic oxidation with electron transfer readout	Detects oxidative stress; rapid signal	Low specificity among ROS types; rapid degradation *in vivo*
pH Sensors (ISFETs, Redox Dyes)	Extracellular pH	Proton-induced potential shifts or redox shifts	Label-free; responsive to metabolic acidosis	Signal drift, temperature, and ionic interference
Fluorescent / Optical Sensors with Bioelectronic Integration	Oxygen, pH	Ratio-metric dye response integrated with electronic or photodetector readout	Spatial resolution; optical + electrical readout	Requires an external light source; photobleaching
Wearable or Flexible Patch Sensors	Lactate, pH, Temp, Cytokines	Enzymatic or redox-based electrochemical transduction	Non-invasive; wearable; continuous monitoring	Shallow sensing depth; low spatial resolution
Implantable Wireless Sensors	Oxygen, pH, Tumor Size	CT-guided amperometry, ultrasonic telemetry	Real-time monitoring in deep tissue; human-scale models	Surgical placement; limited sensor life
